# Important Roles of Cellular MicroRNA miR-155 in Leukemogenesis by Human T-Cell Leukemia Virus Type 1 Infection

**DOI:** 10.5402/2012/978607

**Published:** 2012-09-18

**Authors:** Mariko Tomita

**Affiliations:** Department of Pathology and Oncology, Graduate School of Medical Science, University of the Ryukyus, 207 Uehara, Nishihara, Okinawa 903-0215, Japan

## Abstract

Human T-cell leukemia virus type 1 (HTLV-1) is the pathogen that causes the aggressive and lethal malignancy of CD4+ T-lymphocytes called adult T-cell leukemia/lymphoma (ATLL). MicroRNAs (miRNAs), a class of short, noncoding RNAs, regulate gene expression by targeting mRNAs for translational repression or cleavage. miRNAs are involved in many aspects of cell biology linked with formation of several cancer phenotypes. However, the relation between miRNAs and pathologic implication in ATLL is not well elucidated. Here, we evaluated the roles of cellular miRNAs in ATLL caused by HTLV-1. We found that the expression of miR-155 was increased in HTLV-1-positive T-cell lines. miR-155 expression was enhanced by Tax and binding of transcription factors, NF-**κ**B and AP-1, on the transcription binding sites of miR-155 gene promoter region is important to increase the expression of miR-155 by Tax. Transfection of anti-miR-155 inhibitor, which inhibits the function of miR-155, inhibited the growth of HTLV-1-positive T-cell lines. On the other hand, the growth of HTLV-1-negative T-cell lines was not changed by transfection of anti-miR-155. Forced expression of miR-155 enhanced the growth of HTLV-1-positive T-cell lines. These findings indicate that targeting the functions of miRNAs is a novel approach to the prevention or treatment of ATLL.

## 1. Introduction

Human T-cell leukemia virus type 1 (HTLV-1) is the pathogen that causes adult T-cell leukemia/lymphoma (ATLL), which is a unique malignancy of CD4^+^ T cells [1]. HTLV-1 transforms the infected CD4^+^ T lymphocytes and causes ATLL in 2–4% of infected individuals 50–60 years after infection [1, 2]. There are four subtypes of ATLL: acute, lymphoma, chronic, and smoldering types. Although the median survival time is more than 2 years in the relatively indolent chronic and smoldering types, the prognosis of ATLL in aggressive type, acute and lymphoma types, is very poor with a median survival time of less than 1 year [3]. However, at present, no curative therapy for ATLL has been established. Therefore, identification of good therapeutic targets and development of new therapeutic strategies for ATLL are urgent issues for public health in an endemic area.

The molecular mechanism of HTLV-1-mediated transformation and carcinogenesis is still unknown. Tax oncoprotein which is encoded by HTLV-1 is known as the major viral protein that confers prosurvival and proproliferative properties to infected cells at initial transformation [1]. Previous studies have shown that Tax can immortalize primary human T lymphocytes [4] and induce ATLL like phenotype in transgenic mice [5]. Tax is a 40 kDa transactivator protein and stimulates not only expression of viral genes but also those of cellular genes by interacting with numerous signaling pathways including nuclear factor *κ*B (NF-*κ*B), Janus kinase/signal transducer and activator of transcription, activator protein-1 (AP-1), phosphoinositide 3- kinase/Akt, serum-responsive factor, and the cAMP responsive element-binding protein (CREB) pathways [6, 7].

 MicroRNAs (miRNAs) contain a broad class of small 20–25 nucleotide long endogenous RNAs. It has been shown that miRNAs play significant regulatory roles in gene expression by targeting mRNAs for translational repression or cleavage [8]. By regulation of gene expression, miRNAs are involved in many cellular phenotypes, including cell proliferation, differentiation, cell-cycle regulation, and immune surveillance. Alteration of miRNAs expression is not rare in human cancers and plays critical roles in carcinogenesis or cancer progression by changing the mRNA translation patterns [9, 10]. It has been demonstrated that there are particular miRNAs that associate with the clinical futures of human cancers [11–13]. Therefore, these miRNAs might be useful for diagnostic markers and therapeutic targets of human cancers. 

 Oncogenic viruses such as Kaposi's sarcoma-associated herpes virus (KSHV), Epstein-Barr virus (EBV), human cytomegalovirus, and human immunodeficiency virus generate viral miRNAs [14, 15]. Some of the viral miRNAs disturb both viral and cellular target gene expression, resulting in the inhibition of apoptosis and in the stimulation of cell growth [14, 15]. However, miRNAs which are encoded by HTLV-1 have not been identified. Previously, it has been argued the relation between HTLV-1 infection and regulation of host miRNA expression, suggesting important roles of cellular miRNAs in survival and growth of HTLV-1-positive T cells [16–18]. Hypothetically, dysregulation of cellular miRNAs expression patterns by infection of HTLV-1 may impact the progression of ATLL. 

 Recently, we have performed miRNA expression profiling between HTLV-1-positive and -negative T-cell lines by microarray analysis to identify specific cellular miRNAs that play biological roles in the pathogenesis of ATLL (unpublished data). Through microarray data analysis, miRNAs whose expression levels are changed between HTLV-1-positive and -negative T-cell lines were selected. Among selected miRNAs, we focused on miR-155 because miR-155 has been implicated in normal hematopoiesis, immune response, and also in carcinogenesis of many human cancers [19, 20]. miR-155 is encoded in the *BIC* gene and *BIC* RNA is processed into miR-155, that is, the functional miRNA [21]. It has been reported that miR-155 plays important roles in maturation and differentiation of lymphocytes [22]. Indeed, miR-155 expression is induced in activation of lymphocytes [19, 23]. miR-155 knockout mice have impaired immune functions [22]. Transgenic overexpression of miR-155 in mouse studies has shown that these transgenic mice result in increased frequency of tumor formation [24]. However, the pathological roles of miR-155 in carcinogenesis by HTLV-1 infection are not well understood. Here, we investigated how miR-155 expression levels are regulated by HTLV-1 and possible roles of miR-155 in HTLV-1-positive T-cell growth.

## 2. Material and Methods

### 2.1. Cell Lines

All cell lines used were maintained in RPMI 1640 medium supplemented with 10% FBS (JRS Biosciences, Lenexa, KS, USA), 50 U/mL penicillin, and 50 *μ*g/mL streptomycin (Sigma-Aldrich, St. Louis, MO, USA) and incubated in a humidified incubator at 37°C under 5% CO_2_. Jurkat, MOLT-4, and CCRF-CEM were HTLV-1-negative T-cell lines. JPX-9 is a subclone of Jurkat cells, expressing Tax protein under the control of the metallothionein promoter [25]. MT-2, MT-4, C5/MJ, SLB-1, and HUT-102 were HTLV-1-positive T-cell lines. MT-1 and ED-40515(−) were T-cell lines of leukemic cell origin established from ATLL patients. Tax gene in JPX-9 was induced by treatment with 20 *μ*M of CdCl_2_ as described previously [26].

### 2.2. Chemicals

Cadmium chloride (CdCl_2_) was obtained from Nakarai Tesque Inc. (Kyoto, Japan). Bay11-7082 [(E)-3-(4-Methylphenysulfonyl)-2-propenenenitrile] was purchased from Calbiochem (San Diego, CA, USA). 

### 2.3. Antibodies

The following antibodies were used for western blotting: anti-Tax (1 : 3000; Lt-4)[27], actin (1 : 3000; Lab Vision, Fremont, CA, USA), and horseradish-peroxidase-conjugated anti-mouse IgG antibodies (GE Healthcare). The antibodies used for supershift assay were the following: for NF-*κ*B subunits, p50, p65 (RelA), c-Rel, RelB, and p52, and for AP-1 subunits, c-Fos, FosB, Fra1, Fra2, c-Jun, JunB, and JunD (Santa Cruz Biotechnology, Santa Cruz, CA, USA).

### 2.4. Real-Time RT-PCR

Preparation of the samples for real-time RT-PCR was performed as described previously [28]. MirVana miRNA Isolation Kit (Ambion, Austin, TX, USA) was used for total RNA isolation from the cells. First-strand cDNA was synthesized by TaqMan-MicroRNA Reverse Transcription Kit (Applied Biosystems, Foster City, CA, USA). Real-time RT-PCR for miRNAs (TaqMan microRNA Assays, Applied Biosystems) was done on MX3000p real-time PCR system (Stratagene, La Jolla, CA, USA) as described previously [29]. PCR reactions were performed in triplicates, and the values relative to *RNU48* were analyzed by the 2-ΔΔCt method [30].

### 2.5. Western Blotting

Western blotting analysis was performed as described previously [31]. Cells were lysed in a buffer containing 62.5 mM Tris-HCl, pH 6.8, 2% sodium dodecyl sulfate (SDS), 10% glycerol, 6% 2-mercaptoethanol, and 0.01% bromophenol blue. Equal amounts of samples were exposed to electrophoresis on SDS-polyacrylamide gels followed by transfer to a polyvinylidene difluoride membrane (Millipore, Billerica, MA, USA). The protein expression was analyzed by immunoreactivity with the appropriate antibodies. The bands were visualized by enhanced chemiluminescence Plus reagent (GE Healthcare). 

### 2.6. Plasmids

The expression plasmids for wild-type Tax (WT) and mutants thereof (M22 and 703) were described previously [32, 33]. Wild-type and mutant miR-155 promoter reporter plasmids were described previously [34]. 

### 2.7. Reporter Assay

As previously described [28], appropriate reporter and effecter plasmids were transfected into the cells by MicroPorator MP-100 (Digital Bio Technology, Seoul, Korea). The reference plasmid phRL-TK (Promega, Madison, WI, USA), which contains the *Renilla* luciferase gene under the control of the herpes simplex virus thymidine kinase promoter, was cotransfected to correct for transfection efficiency. The cells were harvested by centrifugation 48 h after transfection, washed with PBS, and lysed in reporter lysis buffer (Promega). Then luciferase activity was analyzed by using the Dual-Luciferase Reporter Assay System (Promega). 

### 2.8. Preparation of Nuclear Proteins

Nuclear proteins were extracted as described previously with some modifications [35]. In brief, cells were washed twice with cold PBS and solubilized in 200 *μ*L lysis buffer A1 (10 mM HEPES, pH 7.9, 10 mM KCl, 0.1 mM EDTA, 0.1 mM EGTA, 1 mM DTT, 2 mM AEBSF) for 10 min at 4°C. Then 200 *μ*L cold lysis buffer A2 (A1 with 0.6% Nonidet P-40 at final concentration) was added. Nuclei were prepared by microcentrifugation for 5 minutes at 4°C. The nuclear pellet was suspended in 75 *μ*L buffer C (20 mM HEPES, pH 7.9, 0.4 M NaCl, 1 mM EDTA, 1 mM EGTA, 1 mM DTT, 2 mM AEBSF, 33 *μ*g/mL aprotinin, 10 *μ*g/mL leupeptin, 10 *μ*g/mL E-64, and 10 *μ*g/mL pepstatin A) and incubated for 30 min at 4°C with brief mixing. The nuclear protein was microcentrifuged (15 000 rpm) for 15 min at 4°C. The protein concentration was measured using the Bradford assay (Bio-Rad, Richmond, CA, USA).

### 2.9. Electrophoretic Mobility Shift Assay (EMSA)

As previously described [36], nuclear proteins (5 *μ*g) were incubated in 20 *μ*L total reaction volume, containing 10 mM Tris-HCl, pH 7.5, 50 mM NaCl, 1 mM EDTA, 1 mM DTT, 5% glycerol, and 1 *μ*g poly-deoxy-inosinic-deoxy-cytidylic acid (GE Healthcare, Waukesha, WI, USA). Then radiolabeled oligonucleotide probes were added and mixtures were incubated for 15 min at room temperature. The probes were prepared by annealing the sense and antisense synthetic oligonucleotides and filling in the overhanging ends in the presence of radiolabeled deoxyadenosine triphosphate and deoxycytidine triphosphate using the Klenow DNA polymerase. The oligonucleotides for the probes are the following: NF-*κ*B binding site of the *miR-155* gene promoter 5′-gactTACTATGGGATTTCCAGCTC-3′ containing a putative NF-*κ*B binding site (underlined), mutant NF-*κ*B binding site 5′-gatcTACTATGCTCGAGCCAGCTC-3′ containing a mutated NF-*κ*B binding site (underlined), AP-1 binding site of the *miR-155* gene promoter 5′-gactcggttatgagtcacaagtga-3′ containing a putative AP-1 binding site (underlined), and mutant AP-1 binding site 5′-gatcCGGTTATCTCGAGCAAGTGA-3′ containing a mutated AP-1 binding site (underlined). The specificities of the DNA-protein complex were determined by adding antibodies specific for NF-*κ*B or AP-1 family proteins to elicit supershifted bands. Antibodies were preincubated with the nuclear proteins for 45 min at room temperature and then incubated with radiolabeled probes. Samples were subjected to electrophoresis on 4% polyacrylamide gels with 0.25 × TBE buffer (22.3 mM Tris, 22.2 mM boric acid, and 0.5 mM EDTA), and the DNA-protein complexes were visualized by autoradiography.

### 2.10. Anti-miRNA Oligonucleotides and miRNA Precursors

Anti-miR-155 oligonucleotide (anti-miR-155 inhibitor) used for miRNA knockdown and the miR-155 precursor (pre-miR-155 precursor) were purchased from Ambion. The cells were transfected with anti-miR-155 inhibitor or pre-miR-155 precursor at final concentration of 50 nM by MicroPorator MP-100. Anti-miR miRNA Inhibitors-Negative Control number 1 and Pre-miR miRNA Precursor Molecules-Negative Control number 1 (Ambion) were used for negative controls. As described previously [28], transfected cells were cultured for 12 h and then plated on 24-well culture dishes at 1 × 10^5^ viable cells/mL. Then, the cells were cultured for additional 24, 48, and 72 h, and the effects of anti-miR-155 inhibitor or pre-miR-155 precursor on the cell growth were determined by counting the number of viable cells in triplicate. The number of the viable cells was counted by the Trypan Blue dye exclusion assay. 

### 2.11. Statistical Analysis

All values were the mean ± SD from three separate experiments. Differences between samples were analyzed by the Student's *t*-test. A *P* value less than 0.05 was considered the presence of a statistically significant difference.

## 3. Results

### 3.1. miR-155 Is Highly Expressed in HLTV-1-Positive T-Cell Lines

**Figure 1:**
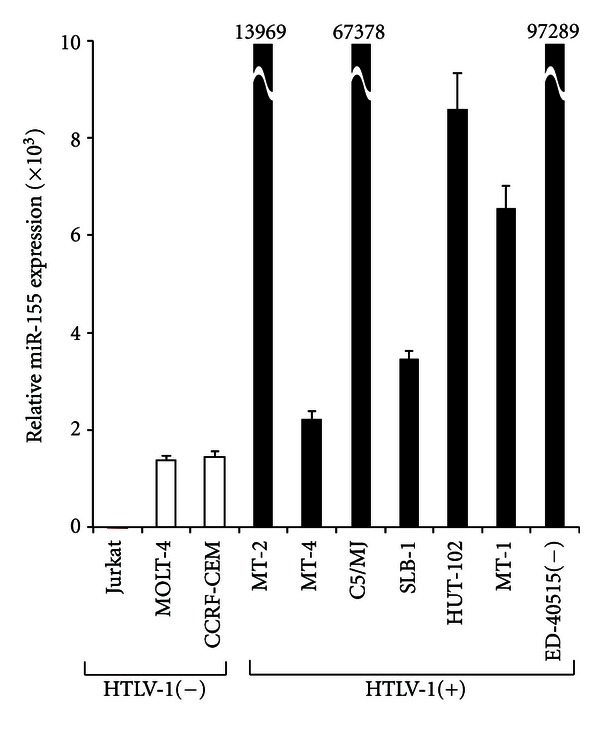
High expression of miR-155 in HTLV-1-positive T-cell lines. The expression of miR-155 in HTLV-1-positive (HTLV-1(+)) and in HTLV-1-negative T-cell lines (HTLV-1(−)) was analyzed by TaqMan real-time RT-PCR. Values are displayed as fold induction of miR-155 expression relative to that in Jurkat cells. Values are the mean ± SD from three separate experiments. Numbers on the MT-2, C5/MJ, and ED-40515(−) represent the real values.

MiRNA expression profiling in HTLV-1-positive T-cell line MT-2 and HLTV-1-negative T-cell line Jurkat were analyzed by microarray (unpublished data). Differentially expressed miRNAs between HTLV-1-positive and -negative T-cells exceeding 2-fold were selected. In this study, we focused on miR-155 among chosen miRNAs because miR-155 has been implicated in lymphocyte activation and in carcinogenesis of many human cancers [19, 20]. Then, we confirmed the high expression of miR-155 in HTLV-1-positive T-cell lines by TaqMan real-time RT-PCR (Figure 1).

### 3.2. miR-155 Expression Is Enhanced by Tax

**Figure 2:**
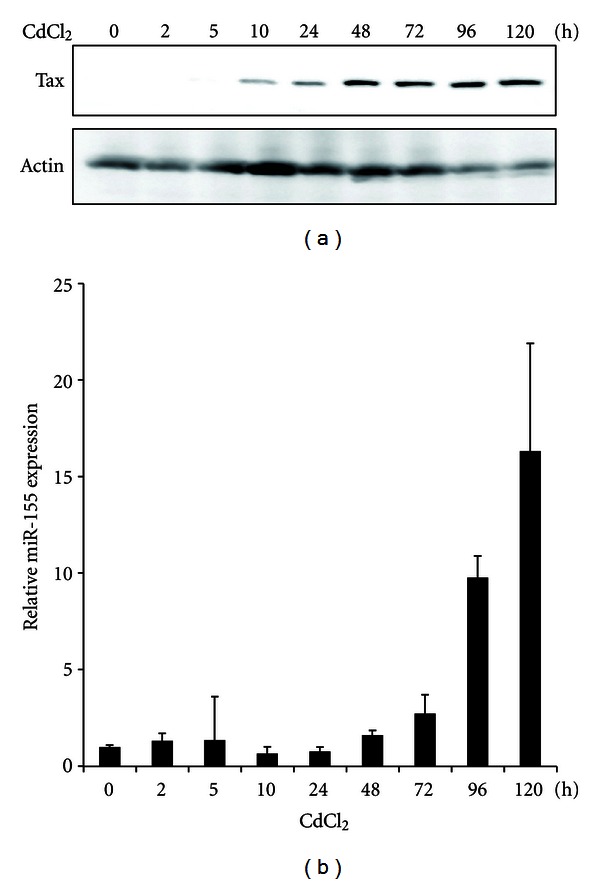
The expression of miR-155 was induced by Tax in T cells. Tax-inducible cell line, JPX-9 cells were cultured with CdCl_2_ (20 *μ*M) for indicated time periods. (a) Tax expression in CdCl_2_-treated JPX-9 cells. Cell lysates were prepared from CdCl_2_-treated JPX-9 cells at the indicated time points. Tax protein expression after CdCl_2_ treatments was determined by western blot. Actin protein expression served as a loading control. (b) miR-155 expression was analyzed by real-time RT-PCR. miR-155 expression is shown as a fold induction relative to the values measured at 0 h. Values are the mean ± SD from three separate experiments.

Next, we determined the role of Tax in miR-155 expression by using Tax-inducible cell line [25]. As we have shown previously [28], we confirmed that JPX-9 cells expressed Tax around 10 h after incubation with CdCl_2_ by western blotting. Tax expression persisted at least until 120 h after induction (Figure 2(a)). Then, we analyzed the expression of miR-155 in the cells by TaqMan real-time RT-PCR. MiR-155 expression in JPX-9 cells treated with CdCl_2_ started increasing from 48 h and constantly increased until 120 h after incubation with CdCl_2_ (Figure 2(b)). MiR-155 expression was not altered by CdCl_2_ in the parental Jurkat cells (data not shown). These findings indicate that the expression of miR-155 is enhanced by Tax in T cells.

### 3.3. miR-155 Promoter Activity Is Stimulated by Tax through Activation of NF-*κ*B and AP-1 Elements

**Figure 3:**
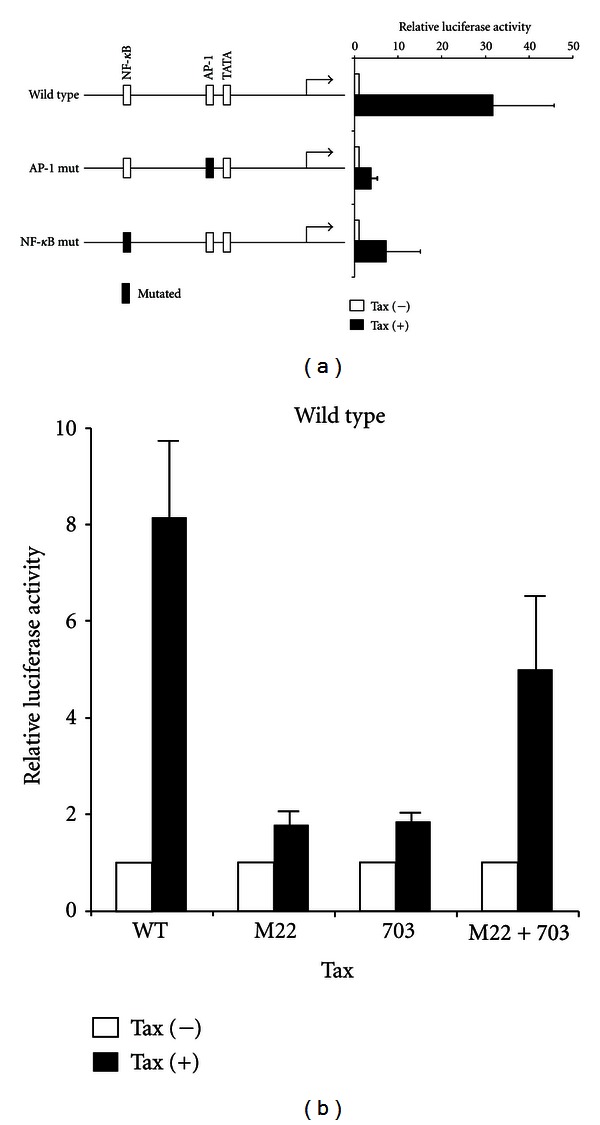
miR-155 promoter activity was increased by Tax through both NF-*κ*B and AP-1 activation. (a) Putative transcription factor binding sites in the miR-155 promoter are presented in the left panel. Luciferase reporter plasmids with either wild-type or mutant *miR-155* gene promoter together with either Tax expression plasmid (Tax (+)) or empty vector (Tax (−)) were transfected into Jurkat cells. Then the cells were incubated for 48 h. Luciferase reporter activity is shown as a fold induction relative to the levels measured in the cells transfected with the empty vector (Tax (−)). (b) Jurkat cells were transfected with following plasmids: luciferase reporter plasmids of wild-type miR-155 promoter together with either Tax wild type (WT), M22, 703 expression plasmids, or empty vector. Luciferase activity was analyzed 48 h later. Values are the mean ± SD from three separate experiments.

To understand transcriptional mechanisms for Tax-induced activation of miR-155 expression, we analyzed the effects of Tax on the transactivation of *miR-155* promoter by luciferase reporter assay. *miR-155* gene promoter region has some high-probability transcription factor binding sites including NF-*κ*B and AP-1 sties [34]. Each of these transcription factor binding sites was mutated in the reporter plasmid (Figure 3(a)). Jurkat cells were transfected with the wild-type or the mutant luciferase reporter plasmid together with or without Tax expression plasmid. Forced expression of Tax enhanced the wild-type reporter activity. In contrast, mutation of the AP-1 site considerably suppressed the Tax-stimulated induction of the miR-155 gene promoter activity, and mutation of NF-*κ*B site also inhibited the effect of Tax on *miR-155* gene promoter activity (Figure 3(a)). However, reduction levels of reporter activity by mutation in NF-*κ*B site was modest than that by mutation in AP-1 site. Tax can activate the transcription of viral and cellular genes through two different enhancers: a cAMP responsive element (CRE)-like sequence and a *κ*B element. Two well-known Tax mutants, Tax M22 and Tax 703, differentially activate these pathways. Tax M22 effectively activated the CRE element but not the NF-*κ*B element. Tax 703 activates the NF-*κ*B element but does not affect CRE [32, 33]. To confirm the transactivation-relevant signaling pathways, these Tax mutants were cotransfected along with the miR-155 promoter construct. In the present study, Tax M22 or 703 slightly activated the miR-155 promoter (Figure 3(b)). On the other hand, transfection with two mutants together could activate miR-155 promoter (Figure 3(b)), indicating that Tax activates the miR-155 promoter in NF-*κ*B- and CRE-dependent manners. However, the miR-155 promoter sequence does not have sequences suggestive of sites for CRE, suggesting that activation of miR-155 promoter by Tax is not directly mediated by the CRE pathways. Previously, it has been shown that Tax mutants M22 and 703 cannot activate AP-1 in a T-cell line and the coexpression of Tax M22 and Tax 703 can activate the AP-1 site [37]. It might be possible that both NF-*κ*B and AP-1 signaling are necessary to achieve full activation of miR-155 promoter by Tax in T-cells.

### 3.4. NF-*κ*B Highly Bound to miR-155 Promoter in HTLV-1-Positive T-Cell Lines

**Figure 4:**
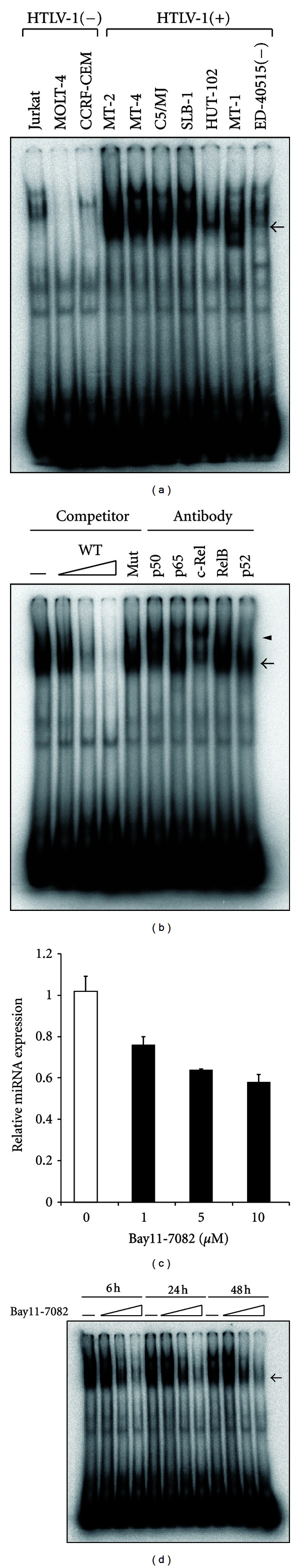
NF-*κ*B signaling is important for miR-155 expression in HTLV-1-positive T cells. (a) DNA-binding activities of proteins to the promoter region of *miR-155* gene in HTLV-1-positive and -negative T-cell lines were evaluated by EMSA. The NF-*κ*B oligonucleotide probe containing the NF-*κ*B-binding site from *miR-155* gene was used. Arrow shows specific DNA-protein complexes. (b) The specificity of NF-*κ*B-DNA binding was analyzed in MT-2 cells. Competition assay with cold competitors of wild type (WT) or mutated (Mut) probe showed the specificity of the protein-DNA-binding complex. Antibodies to NF-*κ*B subunits were used for super shift assay. The arrow shows specific DNA- NF-*κ*B complexes. The supershifted complexes are indicated by an arrowhead. (c) An NF-*κ*B inhibitor, Bay11-7082, inhibited miR-155 expression. Real-time RT-PCR shows miR-155 expression in MT-2 cells treated with different concentration of Bay11-7082. miR-155 expression is demonstrated as a fold induction relative to that in untreated cells. Values are the mean ± SD from three separate experiments. (d) The activity of NF-*κ*B binding to the *miR-155* gene promoter was suppressed by Bay11-7082. MT-2 cells were cultured with 0, 1, 5, or 10 *μ*M of Bay11-7082 for indicated time periods. The NF-*κ*B binding activity on *miR-155* gene promoter was determined by EMSA. Specific DNA-NF-*κ*B subunits complexes are indicated by the arrow.

We next examined NF-*κ*B-miR-155 promoter binding in HTLV-1-positive and -negative T-cell lines by EMSA. The oligonucleotide probe with an NF-*κ*B site of *miR-155* gene promoter was used. Protein-DNA-binding activity was highly detected in HTLV-1-positive T-cell lines. In contrast, those in HTLV-1-negative T-cell lines were weak (Figure 4(a)). Observed protein-DNA complexes in HTLV-1-positive T-cell lines were revealed to be specific for NF-*κ*B by competition assays using cold probes (Figure 4(b)). The complex was supershifted by antibodies specific for NF-*κ*B p50, p65, and c-Rel, indicating that the NF-*κ*B-binding protein complexes contained these NF-*κ*B subunits (Figure 4(b)). These results indicate that NF-*κ*B proteins activate the expression of miR-155 by binding to *miR-155* gene promoter in HTLV-1-positive T-cell lines.

### 3.5. The Expression of miR-155 Is Reduced by NF-*κ*B Inhibitor

Then, we determined the effects of NF-*κ*B inhibition on miR-155 expression in HTLV-1-positive T-cell lines by using NF-*κ*B inhibitor, Bay11-7082. HTLV-1-positive MT-2 cells were incubated with Bay11-7082, and miR-155 expression levels were determined by real-time RT-PCR. miR-155 expression levels were decreased by Bay11-7082 treatment (Figure 4(c)). EMSA demonstrated that Bay11-7082 also suppressed NF-*κ*B-*miR-155* gene promoter binding in HTLV-1-positive T cell (Figure 4(d)). These findings support the idea that NF-*κ*B enhances the expression of miR-155 by stimulation of transcriptional activity of *miR-155* gene.

### 3.6. AP-1 Highly Bound to miR-155 Gene Promoter in HTLV-1-Positive T-Cell Lines

**Figure 5:**
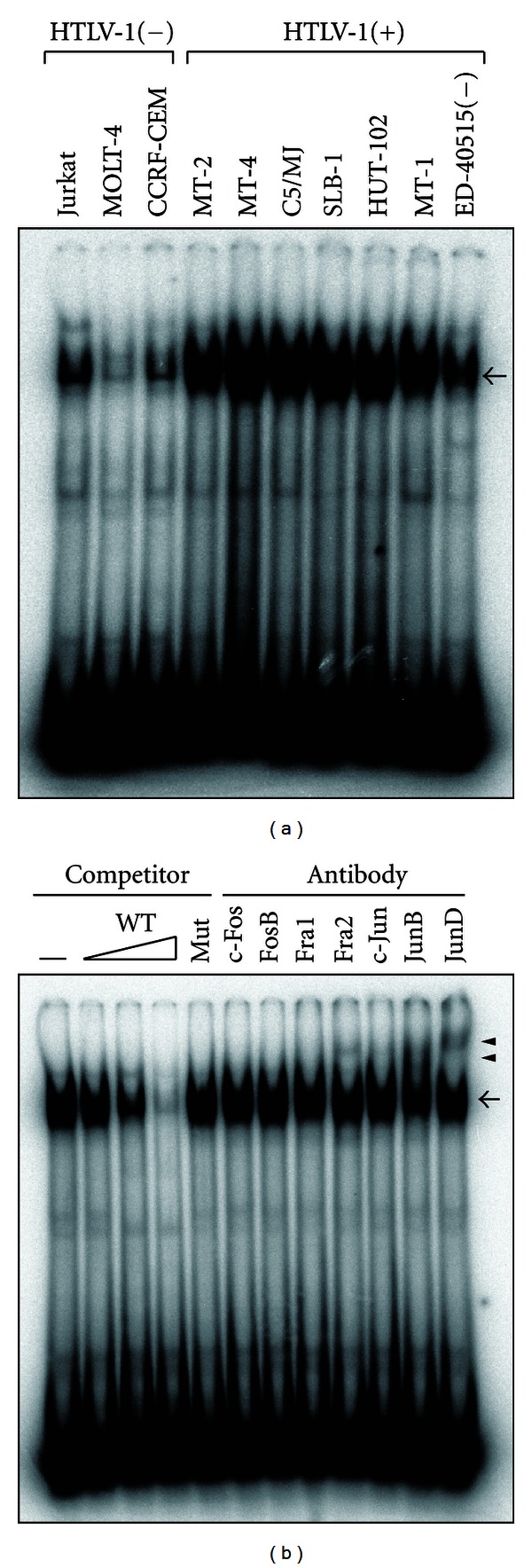
AP-1 signaling is important for miR-155 expression in HTLV-1-positive T cells. (a) DNA-binding activities of proteins to the *miR-155* gene promoter in HTLV-1-positive and -negative T-cell lines was assessed by EMSA. The AP-1 oligonucleotide probe containing the AP-1-binding site from *miR-155* gene was used. Arrow shows specific DNA-protein complexes. (b) The specificity of AP-1-DNA binding was analyzed in MT-2 cells. Competition assay with cold competitors of wild type probe (WT) or mutated probe (Mut) showed the specificity of the protein-DNA-binding complex. Antibodies against various AP-1 subunits were used for supershift assay. Arrow shows specific complexes of AP-1 with AP-1 probes. Arrowheads show supershifted bands.

Then, we analyzed the effects of AP-1 on the expression of *miR-155* gene by EMSA. Like NF-*κ*B, highly AP-1 binding activity was detected in HTLV-1-positive T-cell lines. On the other hand, AP-1 binding activity in HTLV-1-negative T-cell lines was weak (Figure 5(a)). Observed protein-DNA complexes in HTLV-1-positive T-cell lines were revealed to be specific for AP-1 by competition assays using cold probes (Figure 5(b)). The AP-1-binding protein complexes from HTLV-1-positive T-cells included Fra2, JunB, or JunD (Figure 5(b)). These results support the idea that AP-1 proteins activate miR-155 by stimulation of transcriptional activity of *miR-155* gene in HTLV-1-positive T-cell lines.

### 3.7. The Growth of HLTV-1-Infected T-Cell Lines Was Suppressed by Inhibition of miR-155 Function

**Figure 6:**
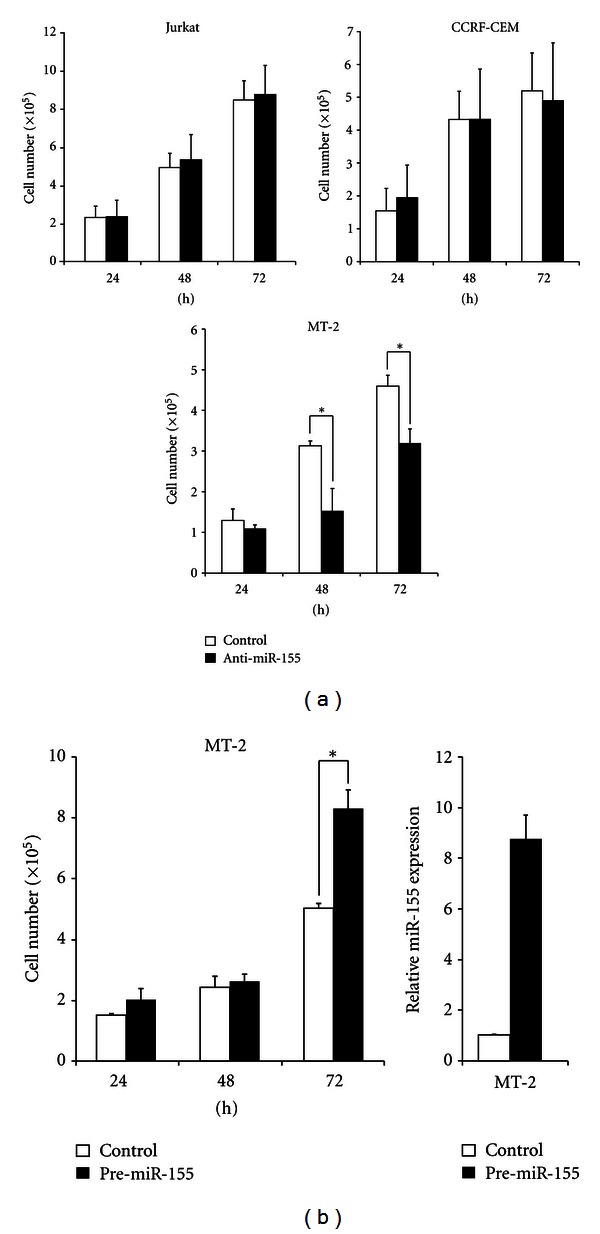
The growth of HLTV-1-infected T cells was suppressed by inhibition of cellular miR-155 function. (a) Anti-miR-155 inhibitor (anti-miR-155) or negative control (control) was transfected into the cells (Jurkat, CCRF-CEM, or MT-2 cells). The growth of the cells was determined by trypan blue dye exclusion assay. (b) Pre-miR-155 precursor (pre-miR-155) or negative control (control) was transfected into MT-2 cells. The growth of the cells was determined by trypan blue dye exclusion assay. Mature miR-155 expression at 72 h was determined by TaqMan real-time RT-PCR. Values are the mean ± SD from three separate experiments (*P < 0.05).

Lastly, to examine the roles of miR-155 in the growth of HTLV-1-positive T-cells, anti-miR-155 inhibitor was transfected into the cells. Anti-miR-155 inhibitor prevents the function of miR-155 specifically. Cell growth was inhibited in HTLV-1-positive MT-2 cells (Figure 6(a), lower panel), but not in HTLV-1-negative Jurkat and CCRF-CEM cells (Figure 6(a), upper panels). In contrast, forced expression of miR-155 by transfection of pre-miR-155 precursor enhanced the growth of MT-2 cells (Figure 6(b), left panel). The expression of mature miR-155 in the cells transfected with pre-miR-155 precursor was confirmed by real-time RT-PCR (Figure 6(b), right panel). These findings indicate that miR-155 specifically improves the growth of HTLV-1-positive T cells.

## 4. Discussion

In this study, we found the stimulatory roles of cellular miR-155 in proliferation of HTLV-1-positive T cells. It has been well known that HTLV-1 Tax activates transcription factors, NF-*κ*B and AP-1 signaling pathways [6, 7]. We showed that these transcription factors bind to the *miR-155* gene promoter, resulting in stimulating the expression of *BIC* gene whose transcripts are processed into miR-155. Indeed, HTLV-1-positive T-cell lines expressed higher levels of miR-155 that HTLV-1-negative cell lines (Figure 1). Overexpression of miR-155 has been shown to correlate with many human cancers [19, 20]. Oncogenic viruses, such as KSHV and Marek's disease virus-1, a herpes virus that causes a lymphoproliferative disorder in chickens, encode their own miR-155 orthologues [38, 39]. For example, miR-K12-11, KSHV viral miRNA, has significant homology with cellular miR-155. Previous studies have shown a correlation between EBV and expression of miR-155 [40]. Although EBV does not encode any miR-155 orthologues, EBV type III latency genes enable high cellular miR-155 expression levels in the EBV-infected cells [34]. Like these oncogenic viruses, our current study linked HTLV-1 infection to high expression of miR-155. In addition to our study, other studies have also demonstrated that cellular miR-155 expression was enhanced in HTLV-1-positive T-cell lines or peripheral blood mononuclear cells from ATLL patients [16–18]. These findings implicate that cellular miR-155 plays crucial role in the biology and pathogenesis of HTLV-1.

Cellular miR-155 expression seems to be regulated by multiple signaling pathways. It has been shown that miR-155 can be induced by B-cell receptor activation through a transcription factor AP-1 site on its promoter region [34]. On the other hand, two other studies have shown that EBV latent protein, LMP1 upregulates miR-155 expression in B cells through an NF-*κ*B-mediated mechanism [41, 42]. In our study, we found that both AP-1 and NF-*κ*B influence expression of *miR-155* gene (Figure 3). Although luciferase assay with reporter plasmids containing mutations on binding sites of each transcription factor revealed that both AP-1 and NF-*κ*B sites are necessary for full activation of miR-155 promoter, effect of mutation on AP-1 site more clearly decreased promoter activity than that of mutation on NF-*κ*B site (Figure 3(a)). These results suggested that AP-1 is more important than NF-*κ*B for activation of *miR-155* gene promoter. Further investigations are needed to conclusively verify which transcription factor is more important for activation of miR-155 promoter activity. 

In this study, we found that overexpressed miR-155 enhances the growth of HTLV-1-positive T cells (Figure 6). Moreover, inhibition of miR-155 functions by anti-miR-155 reduced the growth of these cells. In contrast, this effect of miR-155 in HTLV-1-positive T cells did not work in negative T cells (Figure 6(a)). These observations suggest that miR-155 is one of the important factors which enhanced malignant proliferation of HTLV-1-positive T cells. How does miR-155 play a role in carcinogenesis by HTLV-1-infection? Recent studies have identified many miR-155 targets that may contribute to the modulation of cell proliferation, differentiation, and apoptosis pathways. One of the miR-155 directly targets is the transcript encoding the transcriptional repressor, BACH1, which binds to consensus AP-1 promoter elements and suppresses AP-1 activity [43]. In this study, we found that AP-1 activates miR-155 expression by binding AP-1 site on the promoter of *miR-155* gene (Figures 3 and 5). Considering the known activation of AP-1 by HTLV-1 Tax as well as the constitutive activation of AP-1 in primary ATLL cells [44], it is possible that the suppression of AP-1 inhibitors such as *BTB and CNC homology 1 (BACH1)* by miR-155 may derepress AP-1 promoter activity [45]. In addition, miR-155 can target other transcriptional repressor gene, *human immunodeficiency virus type 1 enhancer binding protein 2 (HIVEP2)* [34]. HIVEP2 protein inhibits the expression of oncogene *c-myc* through an NF-*κ*B binding sequence [46]. It has been shown that HTLV-1 Tax stimulates transactivation of *c-myc* promoter through NF-*κ*B activation [47]. It can be possible that Tax also suppresses *HIVEP2* expression through activation of miR-155 expression and suppression of *HIVEP2* expression may facilitate derepression of NF-*κ*B binding site on *c-myc* promoter in HTLV-1-positive T cells. Further analysis is needed to elucidate which targets are actually inhibited by miR-155 in HTLV-1-positive T cells.

 Using microarray and real-time RT-PCR analysis, we obtained a number of other cellular miRNAs that are also differentially expressed in HTLV-1-positive T-cell lines and primary ATLL cells (unpublished data). Recently, we showed that miR-146a plays a crucial role in the growth of HTLV-1-positive T cells [28]. Similar to miR-155, we demonstrated that miR-146a expression was induced by Tax through NF-*κ*B activation and anti-miR-146a inhibited specifically the proliferation of HTLV-1-positive T-cell lines but not that of -negative T-cell lines [28]. Although Tax plays an important role in overexpression of both miR-155 and miR-146a, other viral proteins and RNAs such as HTLV-1 basic leucine zipper factor might have some functions to modulate cellular miRNA expression in T cell. A recent study has shown that loss of miR-31 is responsible for oncogenic NF-*κ*B activity and malignant phenotypes in ATLL [48]. Not only single miRNA but also combination of several cellular miRNAs which are abnormally expressed in HTLV-1-positive T cells may affect carcinogenesis by HTLV-1 infection. Therefore, the contribution of other cellular miRNAs in carcinogenesis related with HTLV-1 infection must be examined. From this point of view, further investigations might detect new functions of these miRNAs in carcinogenesis by HTLV-1 infection.

## 5. Conclusion

Our study demonstrated that miR-155 is highly expressed in HTLV-1-positive T-cell lines. Tax induced miR-155 expression by activation of NF-*κ*B and AP-1 signaling pathways. Inhibition of miR-155 by anti-miR-155 inhibitor suppressed the growth of HTLV-1-positive T-cell lines. On the other hand, the growth of HTLV-1-negative T-cell lines was not changed by transfection of anti-miR-155. Forced expression of miR-155 increased the growth of HTLV-1-positive T-cell line. Our findings suggest that highly expressed miR-155 inhibit translation of its target mRNAs that enhance the growth of HTLV-1-infected T cells, resulting in progression of ATLL. We propose that, miR-155 is one of the key molecules that are involved in the progression of ATLL.

## Abbreviations

AP-1: Activator protein-1
ATLL: Adult T-cell leukemia/lymphoma
BACH1: BTB and CNC homology 1
CdCl_2_: Cadmium chloride
CRE: cAMP responsive element
CREB: cAMP responsive element-binding protein
EMSA: Electrophoretic mobility shift assay
EBV: Epstein-Barr virus
HIVEP2: Human immunodeficiency virus type 1 enhancer binding protein 2
HTLV-1: Human T-cell leukemia virus type 1
KSHV: Kaposi's sarcoma-associated herpes virus
miRNAs: MicroRNAs
NF-*κ*B: Nuclear factor *κ*B
SDS: Sodium dodecyl sulfate.
